# Automatic joint segmentation and classification of breast ultrasound images via multi-task learning with object contextual attention

**DOI:** 10.3389/fonc.2025.1567577

**Published:** 2025-04-08

**Authors:** Yaling Lu, Fengyuan Sun, Jingyu Wang, Kai Yu

**Affiliations:** ^1^ Department of Medicine Ultrasound, People’s Hospital of Tianfu New Area in Sichuan, Chengdu, Sichuan, China; ^2^ Guangxi Wireless Broadband Communication and Signal Processing Key Laboratory and School of Information and Communication, Guilin University of Electronic Technology, Guilin, Guangxi, China; ^3^ School of Software Engineering, Xi’an Jiaotong University, Xi’an, Shaanxi, China; ^4^ Beijing Smartmore Intelligent Technology Co., Ltd, Digital Intelligence Business Department, Beijing, China

**Keywords:** breast ultrasound images, segmentation, classification, deep learning, multi-task learning

## Abstract

The segmentation and classification of breast ultrasound (BUS) images are crucial for the early diagnosis of breast cancer and remain a key focus in BUS image processing. Numerous machine learning and deep learning algorithms have shown their effectiveness in the segmentation and diagnosis of BUS images. In this work, we propose a multi-task learning network with an object contextual attention module (MTL-OCA) for the segmentation and classification of BUS images. The proposed method utilizes the object contextual attention module to capture pixel-region relationships, enhancing the quality of segmentation masks. For classification, the model leverages high-level features extracted from unenhanced segmentation masks to improve accuracy. Cross-validation on a public BUS dataset demonstrates that MTL-OCA outperforms several current state-of-the-art methods, achieving superior results in both classification and segmentation tasks.

## 1 Introduction

Breast cancer is the most common cancer among women worldwide and the leading cause of cancer-related deaths in females globally (1–3). Therefore, early diagnosis of breast cancer is crucial for reducing mortality, as more than 90% of cases can now be detected at an early stage and treated before becoming metastatic (4). Among these technologies, ultrasound imaging (USI) is one of the most widely used screening methods for early breast cancer detection due to its high convenience, low cost, and high effectiveness. USI uses sound waves to generate images of the body’s internal structures, which are subsequently analyzed by computer-aided diagnosis (CAD) systems to enhance diagnostic accuracy (5). Automated segmentation and classification are critical steps in CAD systems for interpreting breast ultrasound (BUS) images, forming a foundation for further analysis and treatment planning. In recent years, various image processing tools have been developed for detecting and delineating affected areas in ultrasound images, including segmentation, enhancement, and classification techniques (6–8).

With advancements in computer technology, various machine learning (ML) methods have been proposed Lu et al. (9)Liu et al. (10) and successfully applied to medical image processing and interpretation (11–13). ML methods have also shown great potential in the segmentation and classification of breast ultrasound (BUS) images (14–16). Techniques such as k-nearest neighbors (17), support vector machines (18), and clustering algorithms (19). For example, da Silva et al. applied several ML-based methods, including multilayer perceptrons, k-nearest neighbors, and support vector machines, for breast cancer detection and diagnosis, and compared their performance in BUS image segmentation and classification (20). Lyu and Cheung proposed a hierarchical extreme learning machine (H-ELM) for efficient breast cancer ultrasound analysis (21). Zhu et al. employed random forest regression for the automatic measurement of fetal femur length in ultrasound images (22) and compared their approach with the widely used convolutional neural network (CNN) model, SegNet (23).

Recently, state-of-the-art deep learning algorithms have been proposed for various classification and segmentation tasks (24–28). These algorithms include convolutional neural networks (CNNs) (29, 30), Transformer (31–33), quantum-enhanced deep learning (34–36), and ensemble learning (37, 38). Among these algorithms, CNNs are some of the most widely used methods that is capable of building strong non-linear relationships between training data and labels (39–41). CNN-based architectures have been successfully applied in medical image processing (42–44) and have been employed for interpreting breast ultrasound (BUS) images (45, 46). For instance, Deawon et al. compared the performance of various models in diagnosing breast cancer (47), including image classification models (VGGNet19, ResNet50, DenseNet121, EfficietNet v2) and image segmentation models (UNet, ResUNet++, DeepLab v3). Other notable deep learning algorithms for processing BUS images include generative adversarial networks (GANs) (48), edge enhanced model (49), and probability-based optimal deep learning (50). However, many of these methods follow a two-step approach: first segmenting the BUS images and then classifying them. This two-step approach results in lower computational efficiency and less accurate outcomes. To address these challenges, multi-task learning (MTL) has been introduced, enabling the joint segmentation and classification of BUS images within a unified, end-to-end framework (51, 52). Despite this advancement, most existing MTL methods rely on low-level features learned from the down-sampling path of the network (e.g., UNet’s encoder), which limits their ability to capture high-level semantic information. MTL-COSA utilizes segmentation masks to guide the classification task (53). However, MTL-COSA lacks a segmentation enhancement module to further improve performance.

In this study, we present a novel multi-task learning network (MTL-OCA) that integrates an object contextual attention module for simultaneous segmenting and classifying breast ultrasound (BUS) images. Our key contributions are as follows:

- We propose an innovative multi-task learning architecture that simultaneously handles segmentation and classification tasks for BUS images.
- We introduce an object contextual attention module that refines segmentation masks by learning pixel-region relationships. The high-level features extracted from unenhanced segmentation masks are used to enhance the classification performance.
- Our experimental results demonstrate that MTL-OCA outperforms several state-of-the-art methods in both segmentation and classification tasks.

The remainder of this paper is organized as follows: Section 2 introduces the deep learning framework, loss functions, and evaluation metrics; Section 3 presents the experimental results and analysis; Section 4 discusses the strengths and limitations of our approach; and Section 5 concludes the paper.

## 2 Methodology

**Figure 1:**
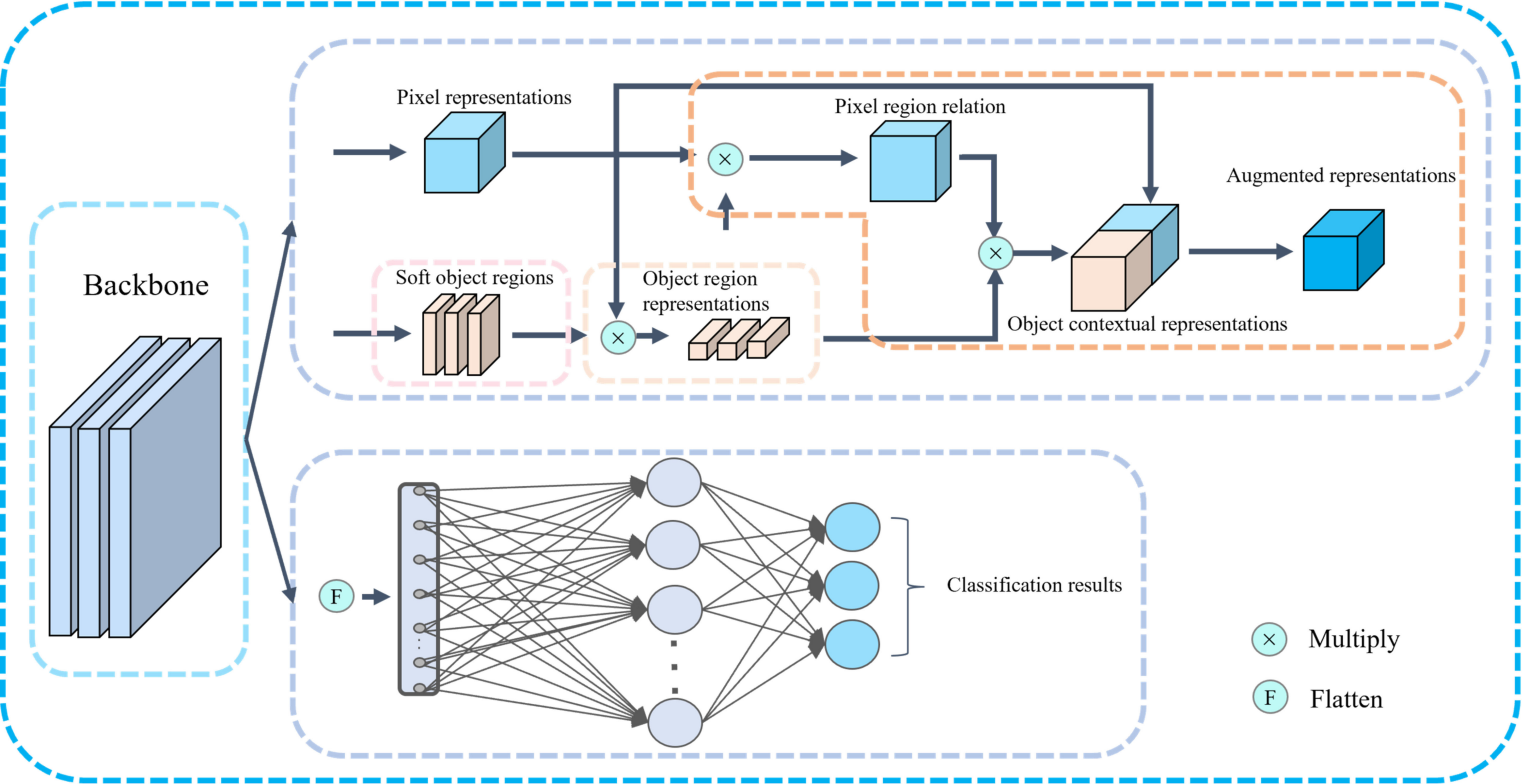
Framework of the proposed MTL-OCA Model. The black arrows present the data flows.

**Figure 1** illustrates the architecture of our proposed multi-task learning network (MTL-OCA). MTL-OCA uses Res-UNet as its backbone to learn pixel-region relationships, which enables the generation of highly accurate segmentation masks. The network processes batches of 2D BUS images and produces both segmentation masks and one-hot encoded classification results.

### 2.1 Overview of our model

**Figure 2:**
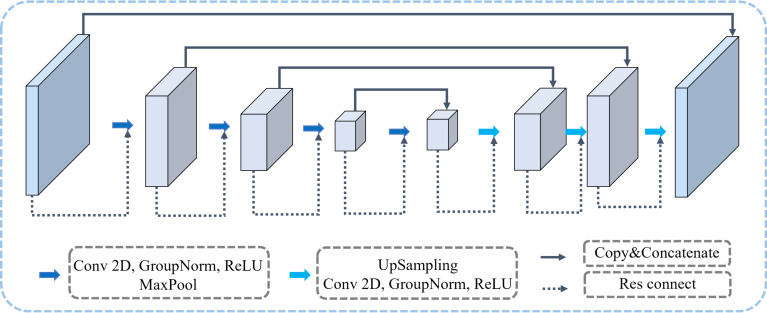
The backbone of our proposed MTL-OCA model. Each rectangular block represents the network’s feature map at varying depths.

**Figure 1** illustrates the architecture of our proposed MTL-OCA, which simultaneously predicts segmentation masks and classification results. The network comprises three main components: the backbone, the object contextual attention module for segmentation, and the classification module for image classification. The backbone is built on a widely used Res-UNet (54), which effectively extracts feature maps from the input images. This Res-UNet employs a three-layer UNet architecture with residual connections, where both the encoder and its symmetric decoder consist of three layers of convolutional blocks (**Figure 2**). Each block in the encoder includes a Conv2D layer, a GroupNorm layer, and a MaxPooling layer for feature extraction and down-sampling. Similarly, each block in the decoder comprises an Upsample layer, a Conv2D layer, and a GroupNorm layer. Unlike previous multi-task learning approaches that typically rely on features extracted solely from the encoder for classification, our model leverages the complete feature maps from the backbone for both segmentation and classification tasks, thereby enhancing performance in BUS image segmentation and classification.

The feature maps extracted from the backbone serve as inputs to the object contextual attention module that learns pixel-region relationships to generate enhanced segmentation masks. As illustrated in **Figure 1**, the contextual pixels are partitioned into *K* soft object regions, and each corresponds to one of the *K* segmentation classes. Each soft object region quantifies the degree to which a given pixel belongs to class *k*, denoted as *d_k_* (with *K* set to 2 in this study). These soft object regions are learned in a supervised manner and essentially serve as coarse segmentation masks. Subsequently, the pixel representations (i.e., the feature maps from the backbone) are multiplied by the soft object regions to produce the object region representations, defined as


(1) $$f_{k}=\sum_{i\epsilon I}d_{ki}x_{i},$$

where *I* denotes the set of pixels in the feature maps. *x_i_* represents the feature of pixel *p_i_* and $d_{ki}$ quantifies the degree to which pixel *p_i_* belongs to the *k^th^* object region. We obtain the pixel-region relation by multiplying the pixel representations by their corresponding soft object regions as follows


(2) $$w_{ik}=\frac{e^{l\left(x_{i},f_{k}\right)}}{\sum_{j=1}^{K}e^{l\left(x_{i},f_{j}\right)}},$$

where l(x,f) denotes the nonlinear transformation implemented by the convolutional blocks. Subsequently, the object regions corresponding to the same class are multiplied to enhance the contextual representations, expressed as


(3) $$y_{i}=\text{ð}\left(\sum_{k=1}^{K}w_{ik}ℋ\left(f_{k}\right)\right),$$

where ð(·) and ℋ(·) denote nonlinear transformations implemented by convolutional blocks. Finally, the augmented representations, which produce accurate segmentation masks, are generated by concatenating the contextual and pixel representations. Equation 1 computes the object region representations, Equation 2 establishes the pixel region relations, and their combined outputs serve as inputs to Equation 3 for generating contextual representations. Meanwhile, the classification module performs the classification task on the same feature maps extracted from the backbone. These features, which are derived from the backbone rather than solely from the encoder, contain richer high-level segmentation semantics that guide the classification process. As indicated in the lower half of **Figure 1**, the classification module is implemented using two layers of a multilayer perceptron (MLP). In this work, the classification results are categorized into three classes: benign, malignant, and normal. So, the final layer consists of three neurons.

### 2.2 Loss function and implementation details

Since the proposed network is designed to perform both segmentation and classification simultaneously, the overall loss function is formulated as a combination of the segmentation loss and the classification loss as follows


(4) $$ℒ={ℒ}_{s}+{ℒ}_{c},$$

where ${ℒ}_{s}$ and ${ℒ}_{c}$ represent the loss functions for the segmentation and classification tasks, respectively. Moreover, the segmentation loss is formulated as a weighted sum of the losses computed on the soft object regions and the augmented representations, written as


(5) $${ℒ}_{s}=\alpha {ℒ}_{soft}+{ℒ}_{aug},$$

where, ${ℒ}_{soft}$ and ${ℒ}_{aug}$ are used to supervise the soft object regions and the augmented representations, respectively. *α* serves as the weighting factor and is set as 0.4 according to the discussions in (55). All the loss functions described in Equations 4, 5 are implemented as cross-entropy.

All deep learning neural networks in this study were implemented and trained on a computer configured with CUDA 11.4, Python 3.7, and PyTorch 1.11, along with other essential libraries. The computer’s hardware setup comprised an Intel 16-core processor, 256 GB of system memory, and two NVIDIA RTX 3090 GPUs (each with 24 GB of dedicated memory). Our MTL-OCA network was trained on the training dataset for 400 epochs with a batch size of 16. Adam optimizer with an initial learning rate of 1*e* ^−3^ is adopted in the training process.

## 3 Results and discussions

### 3.1 Data introduction and preparation

In this study, we employ two BUS image datasets to evaluate the effectiveness of our proposed model. The first dataset, OASBUD dataset (56), comprises 780 BUS images collected from 600 women aged between 25 and 75. These images are classified into three categories: normal, benign, and malignant (**Table 1**). The second dataset, UDIAT (57), which consists of 163 ultrasound images, including 110 benign and 53 malignant cases. Due to different image sizes, we resize all the images to 128 × 128 pixels.

**Table 1:** Three classes of breast cases and the number of images in each category of the OASBUD dataset.

Categories	Image number
Benign	437
Malignant	210
Normal	133
Total	780

Note that all input images in both datasets, excluding the labels, are three-channel, which increases the computational cost and load. To reduce this complexity, we convert the BUS images to grayscale prior to the training process. Additionally, we perform 5-fold cross-validation on the datasets. We further augment the training data through various image transformations, including random rotations (ranging from -45° to 45°), random flips, and random center cropping with a 50% probability. Moreover, we apply image contrast enhancement techniques to enrich our dataset, enabling the network to better handle images with varying contrast levels.

### 3.2 Data pre-processing

To enhance the segmentation results at tumor boundaries, we incorporate the Gaussian derivatives of BUS images, which highlight the edges of tumor boundaries. Let *f* (*x,y*) represent a BUS image. The 2D Gaussian kernel function is defined as


(6) $$G_{2\text{D}}\left(x,y;\sigma \right)=\frac{1}{2\pi \sigma^{2}}e^{-\frac{x^{2}+y^{2}}{2\sigma^{2}}},$$

where σ is the standard deviation that determines the width of the Gaussian kernel function. Then Gaussian derivative of BUS images is defined as


(7) $$\begin{array}{l} g_{\text{x}}=\frac{\partial \left(f*G_{2\text{D}}\right)}{\partial x},\\ g_{\text{y}}=\frac{\partial \left(f*G_{2\text{D}}\right)}{\partial y}. \end{array}$$

The derivative of the Gaussian kernel function respective to x and y can be expressed as


(8) $$\begin{array}{l} \frac{\partial \left(G_{2\text{D}}\right)}{\partial x}=\frac{-x}{2\pi \sigma^{4}}e^{-\frac{x^{2}+y^{2}}{2\sigma^{2}}},\\ \frac{\partial \left(G_{2\text{D}}\right)}{\partial y}=\frac{-y}{2\pi \sigma^{4}}e^{-\frac{x^{2}+y^{2}}{2\sigma^{2}}}. \end{array}$$

The magnitude of the Gaussian derivative of the BUS image is given by


(9) $$\begin{array}{l} m=\sqrt{g_{\text{x}}^{2}+g_{\text{y}}^{2}}. \end{array}$$

**Figure 3:**
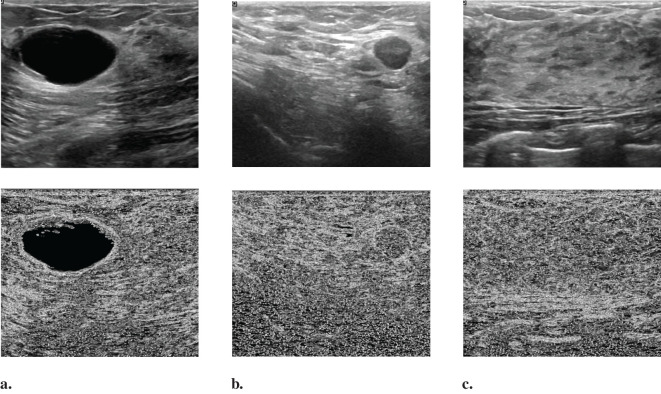
Randomly selected images from the OASBUD dataset and their corresponding Gaussian derivative magnitude images: **(a)** A representative BUS image with a tumor at an early stage, followed by its Gaussian derivative magnitude image; **(b)** A representative BUS image with a malignant tumor, followed by its Gaussian derivative magnitude image; **(c)** A representative BUS image without a tumor, followed by its Gaussian derivative magnitude image.

**Figure 3** displays several BUS images selected from OASBUD detaset. The first row of **Figures 3a, b** show BUS images featuring an early-stage tumor and a malignant tumor, respectively. **Figure 3c** shows a normal BUS image. Their corresponding Gaussian derivative magnitude images, calculated using (Equations 6–9), are presented in the second row of **Figures 3a-c**. Note that the Gaussian derivatives enhance the distinctive features of these BUS images. Notably, we use the computed magnitude as a boundary feature and concatenate it with the original image to create a new input for the network, thereby improving overall task performance.

### 3.3 Results and discussions

To validate the effectiveness of our proposed model, we compare it against two widely used methods: UNet (58) and MT-DUNet (59). UNet is a popular method for both segmentation and classification tasks, while MT-DUNet is a well-established multi-task learning model specifically designed for BUS image segmentation and classification.

**Figure 4:**
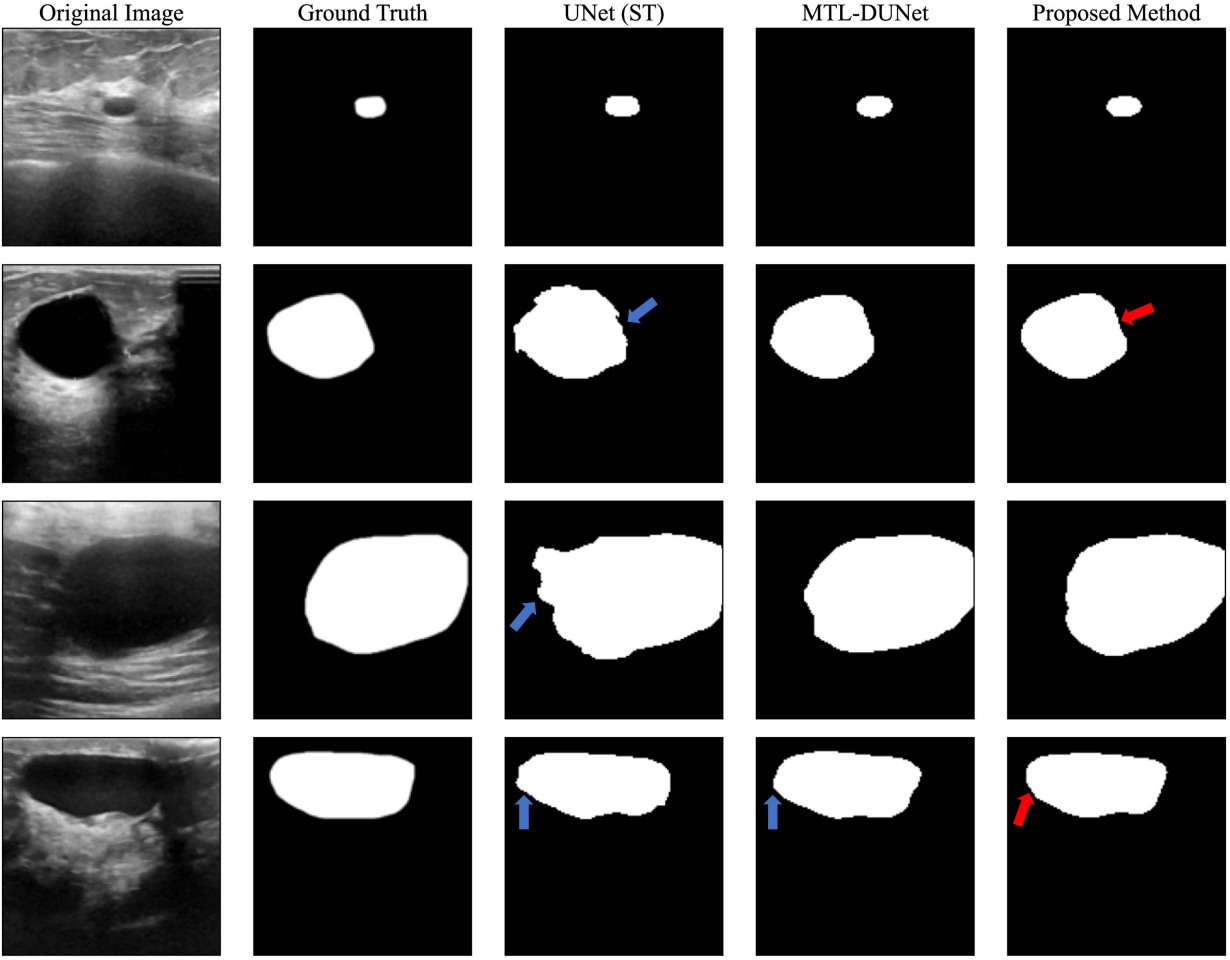
Segmentation results for randomly selected images from the OASBUD dataset using different methods. The first and second columns show the original BUS images and the ground truth labels. The third, fourth, and fifth columns display the segmentation results obtained using UNet, MT-DUNet, and our proposed MTL-OCA, respectively. The blue arrows highlight areas with inaccurate interpretations, whereas the red arrows indicate challenging regions that have been accurately predicted.

After training our models on the OASBUD dataset, we applied all three models to a blind testing dataset. We randomly selected several images from the blind testing dataset and performed image segmentation using each model (**Figure 4**). The blue arrows in **Figure 4** indicate areas with inaccurate prediction while the red arrows point to regions that are challenging to interpret yet accurately predicted. **Figure 4** demonstrates that both our method and MTL-DUNet are more robust than the single-task UNet (denoted as UNet (ST)). This improvement is likely due to the shared features between segmentation and classification tasks (59). We cannot observe the obvious difference between the segmentation results of MTL-OCA and MTL-DUNet. However, their evaluation metrics differ from each other (**Table 2**). Compared to MTLDUNet, the classification module of MTL-OCA improves classification accuracy by fully leveraging the high-level semantic information from the rough segmentation masks.

**Table 2:** The segmentation and classification results on the OASBUD dataset.

Methods	*Dice (%)*	*IoU (%)*	*Acc (%)*
UNet (ST)	77.92	63.83	89.74
MTL-DUNet	79.42	65.86	87.18
Proposed Method	83.75	72.03	91.67

Bold values in Tables indicate the bestperforming results.

**Figure 5:**
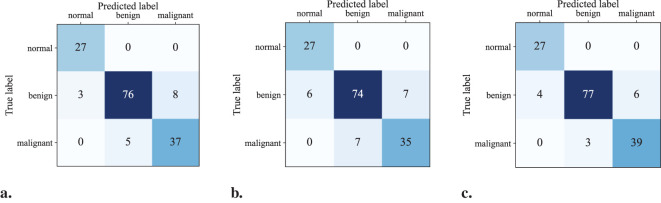
The confusion matrix of the classification results computed using **(a)** UNet(ST), **(b)** MTL-DUNet, and **(c)** our suggested method.

**Figure 6:**
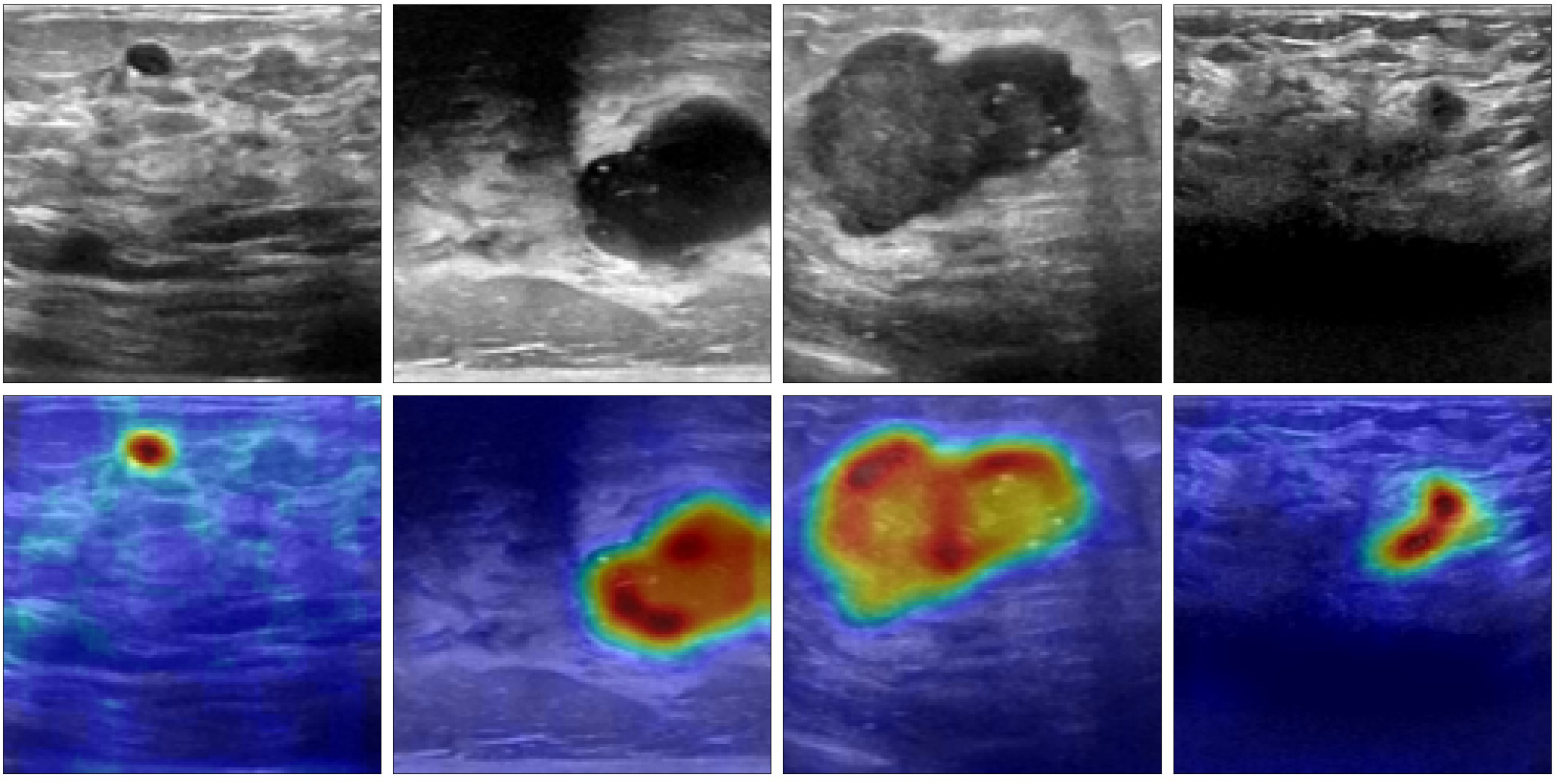
The Grad-CAM heatmaps generated by the proposed method. The first row is the input images and the second row is the corresponding Grad-CAM heatmaps.

**Figures 5a-c** show the confusion matrices for the classification results computed using UNet (ST), MTL-DUNet, and our proposed method, respectively. Note that all models accurately classify the normal class. However, the benign and malignant classification accuracies of our method are notably higher than UNet (ST) and MTL-DUNet. **Table 3** summarizes the *Precision*, *Sensitivity*, *Specificity*, and *F1-score* for these three methods. **Table 3** demonstrates that the proposed method outperforms comparative approaches across all evaluation metrics except for those in normal cases. These quantitative parameters indicate the superiority of our model over both UNet (ST) and MTL-DUNet for this classification task. Additionally, to enhance the interpretability of our model, we generated gradient-weighted class activation mapping (Grad-CAM) heatmaps (**Figure 6**). The highlighted regions in these heatmaps correspond well with the key areas of interest in the input images during classification.

**Table 3:** The *Precision*, *Sensitivity*, *Specificity*, and *F1-score* of UNet (ST), MTL-DUNet, and MTL-OCA on the OASBUD dataset.

Label	UNet (ST)/MTL-DUNet/MTL-OCA			
	*Precision*	*Sensitivity*	*Specificity*	*F1-score*
Normal	0.9/0.818/0.871	1.0/1.0/1.0	0.977/0.954/0.969	0.947/0.899/0.931
Benign	0.938/0.914/0.963	0.874/0.851/0.885	0.928/0.899/0.957	0.905/0.881/0.922
Malignant	0.822/0.833/0.867	0.881/0.833/0.929	0.930/0.939/0.947	0.850/0.833/0.896

Bold values in Tables indicate the best performing results.

**Figure 7:**
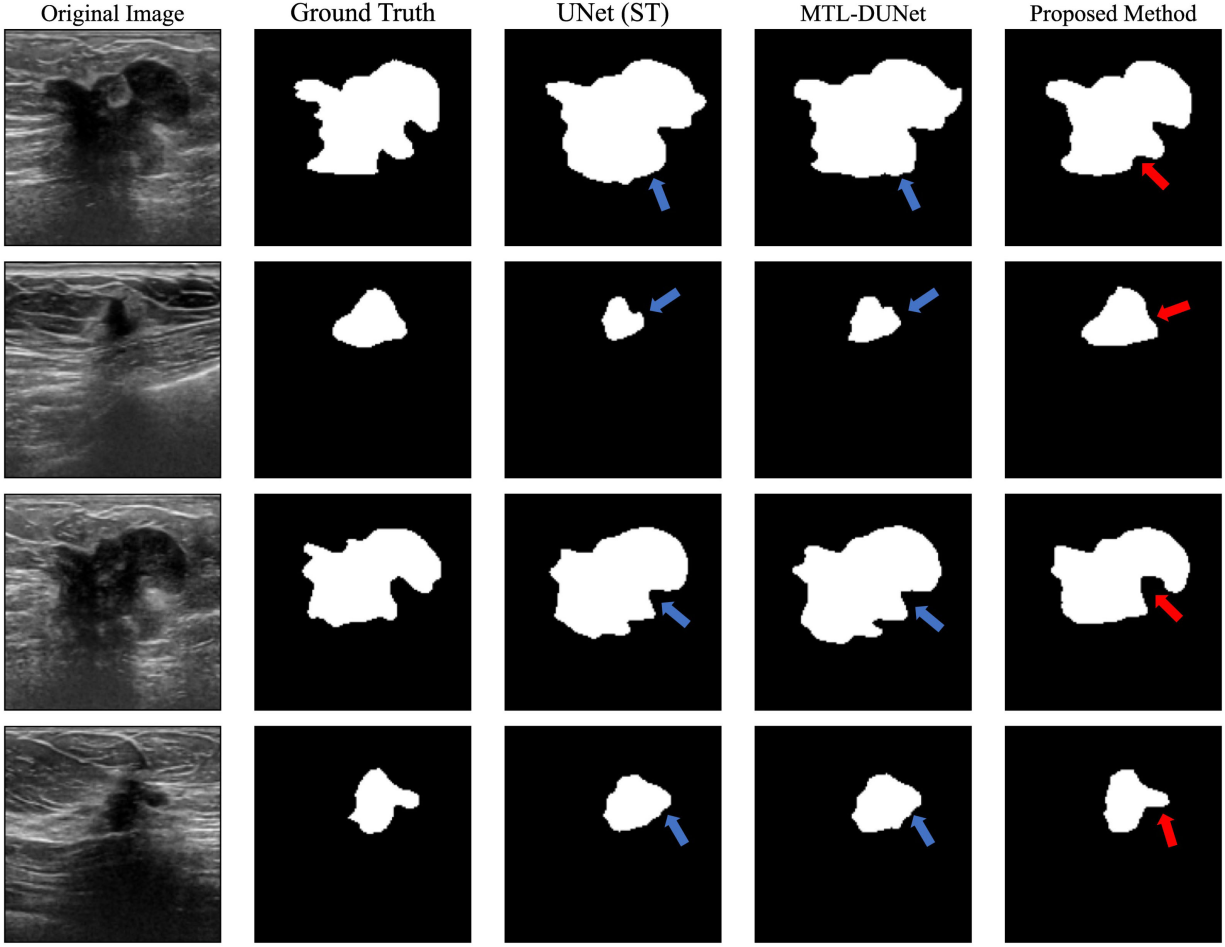
The segmented results for the randomly selected images from the UDIAT dataset using different methods. The first and second columns are the original BUS images and ground truth labels, while the third, fourth, and fifth columns are the segmented results computed using UNet, MTL-DUNet, and our MTL-OCA. The blue arrows highlight areas with inaccurate interpretations, while the red arrows point to regions that are challenging to interpret but have been accurately predicted.

In addition, we trained and tested our model on the UDIAT dataset. The first and second columns in **Figure 7** show randomly selected BUS images and their corresponding ground truth labels, respectively. The third, fourth, and fifth columns show the segmentation results that are computed using different methods. The blue arrows indicate areas with inaccurate interpretations, and the red arrows point to regions that are challenging to interpret but have been accurately predicted. A visual comparison of these results demonstrates that our proposed method achieves superior segmentation performance compared to UNet and MTL-DUNet, particularly at the boundaries highlighted by the blue and red arrows. Furthermore, we evaluated our method on the UDIAT dataset using the same set of metrics (**Tables 4**, **5**). The experimental findings consistently confirm that our approach outperforms the selected comparative methods across all key indicators.

**Table 4:** The segmentation and classification results on the UDIAT dataset.

Methods	*Dice (%)*	*IoU (%)*	*Acc (%)*
UNet (ST)	81.37	68.57	91.33
MTL-DUNet	80.64	67.55	89.29
Proposed Method	84.85	73.68	94.79

Bold values in Tables indicate the best performing results.

**Table 5:** The *Precision*, *Sensitivity*, *Specificity*, and *F1-score* of UNet (ST), MTL-DUNet, and MTL-OCA on the UDIAT dataset.

Label	UNet (ST)/MTL-DUNet/MTL-OCA			
	*Precision*	*Sensitivity*	*Specificity*	*F1-score*
Benign	0.929/0.877/0.958	0.946/0.846/0.964	0.849/0.796/0.913	0.937/0.860/0.961
Malignant	0.872/0.918/0.931	0.698/0.896/0.943	0.953/0.852/0.962	0.774/0.907/0.937

Bold values in Tables indicate the best performing results.

### 3.4 Ablation study

Based on the OASBUD dataset, we conduct an ablation study on the object contextual attention (OCA) module to evaluate its impact on segmentation and classification performance. We test three different configurations. The first configuration omits the OCA module, which means the backbone generates the final segmentation map. As a result, the classification task relies solely on the results from this segmentation. The second configuration includes the OCA module, and the classification task is based on the enhanced segmentation map produced by the OCA. The third configuration is our proposed architecture in this study. **Table 6** presents the results of the ablation study for these three different network architectures when applied to the same testing dataset. **Table 6** indicates that integrating the OCA module improves segmentation performance in the second configuration compared to the first. The result of the third configuration shows that both segmentation and classification performance are inferior to our method when the classification model is based solely on the OCA-enhanced segmentation maps. Overall, the proposed MTL-OCA architecture yields the highest Dice coefficient and classification accuracy. The highest accuracy demonstrates that the optimal integration of the OCA module leads to significant performance enhancement.

**Table 6:** The ablation study on the testing dataset of the OASBUD dataset.

Categories	*Dice (%)*	*Acc (%)*
Model without OCA	75.89	86.15
Model with OCA, classification based on OCA	80.01	90.37
Model with OCA, classification based on backbone	83.75	91.67

Bold values in Tables indicate the best performing results.

## 4 Discussions

The qualitative analysis of the results validates the effectiveness of our proposed multi-task deep learning architecture for the automatic joint segmentation and classification of BUS images. However, we believe that special attention should be paid to three key aspects of the implementation. First, the choice of backbone is crucial for the successful application of a multi-task learning model. In our research, we selected the widely used ResUNet as the backbone. While we have not gone into extensive detail on this choice, prior research has emphasized the importance of selecting suitable neural network architecture. Several advanced models that address various challenges could also serve as viable options for the backbone. Exploring alternative architecture presents a promising avenue for future work. Second, to address the class imbalance in the dataset, we employed data augmentation techniques to modify the class distribution in the training set. For future studies, we may consider using specialized loss functions, such as focal loss (60), to mitigate the issue of class imbalance more effectively. Lastly, while our model performs well on the two datasets used in this study, its performance may be constrained by the size and variety of the training data. Future research will focus on expanding and diversifying the dataset to improve the robustness and generalizability of our approach.

## 5 Conclusions

We propose a multi-task learning network with an object contextual attention module (MTL-OCA) for simultaneous segmentation and classification of BUS images. Numerical experiments on two widely used datasets, namely OASBUD and UDIAT, demonstrate that the object contextual attention module enhances segmentation masks by effectively learning the pixel-region relationships within BUS images. Additionally, the Grad-CAM heatmaps demonstrate that high-level information extracted from the unenhanced segmentation masks can improve classification performance. The comparisons with UNet and MT-DUNet confirm the effectiveness of our model for the joint segmentation and classification of BUS images.

## Data Availability

Publicly available datasets were analyzed in this study. This data can be found here: https://github.com/hugofigueiras/Breast-Cancer-Imaging-Datasets/tree/main.
